# Neuroprotective Effects of Mitochondria-Targeted Plastoquinone in a Rat Model of Neonatal Hypoxic–Ischemic Brain Injury

**DOI:** 10.3390/molecules23081871

**Published:** 2018-07-27

**Authors:** Denis N. Silachev, Egor Y. Plotnikov, Irina B. Pevzner, Ljubava D. Zorova, Anastasia V. Balakireva, Mikhail V. Gulyaev, Yury A. Pirogov, Vladimir P. Skulachev, Dmitry B. Zorov

**Affiliations:** 1 A. N. Belozersky Institute of Physico-Chemical Biology, Lomonosov Moscow State University, 119991 Moscow, Russia; silachevdn@genebee.msu.ru (D.N.S.); plotnikov@belozersky.msu.ru (E.Y.P); irinapevzner@mail.ru (I.B.P.); lju_2003@list.ru (L.D.Z.); skulach@genebee.msu.ru (V.P.S.); 2 Institute of Molecular Medicine, Sechenov First Moscow State Medical University, 119991 Moscow, Russia; balakireva.anastacia@gmail.com; 3 Faculty of Fundamental Medicine, Lomonosov Moscow State University, Lomonosovsky Prospekt, house 31-5, 117192 Moscow, Russia; mihon-epsilon@yandex.ru; 4 Faculty of Physics, Lomonosov Moscow State University, Leninskye gory, house 1, building 2, 119991 Moscow, Russia; yupi937@gmail.com

**Keywords:** hypoxia–ischemia, mitochondria, mitochondria-targeted antioxidants, plastoquinone, oxidative stress

## Abstract

Neonatal hypoxia–ischemia is one of the main causes of mortality and disability of newborns. To study the mechanisms of neonatal brain cell damage, we used a model of neonatal hypoxia–ischemia in seven-day-old rats, by annealing of the common carotid artery with subsequent hypoxia of 8% oxygen. We demonstrate that neonatal hypoxia–ischemia causes mitochondrial dysfunction associated with high production of reactive oxygen species, which leads to oxidative stress. Targeted delivery of antioxidants to the mitochondria can be an effective therapeutic approach to treat the deleterious effects of brain hypoxia–ischemia. We explored the neuroprotective properties of the mitochondria-targeted antioxidant SkQR1, which is the conjugate of a plant plastoquinone and a penetrating cation, rhodamine 19. Being introduced before or immediately after hypoxia–ischemia, SkQR1 affords neuroprotection as judged by the diminished brain damage and recovery of long-term neurological functions. Using vital sections of the brain, SkQR1 has been shown to reduce the development of oxidative stress. Thus, the mitochondrial-targeted antioxidant derived from plant plastoquinone can effectively protect the brain of newborns both in pre-ischemic and post-stroke conditions, making it a promising candidate for further clinical studies.

## 1. Introduction

Hypoxic–ischemic encephalopathy (HIE) is a leading cause of morbidity and mortality: more than 50% of affected infants by two years of age either die or acquire adverse neurological events such as motor impairment, cerebral palsy, mental retardation, epilepsy, visual and hearing impairment, attention deficits, and hyperactivity [1,2]. The majority of the underlying complications of HIE are a result of impaired cerebral blood flow and oxygen delivery to the brain followed by pathological reactive oxygen species (ROS) production by mitochondria, which is considered one of the key factors of neural cell damage and death [3,4,5]. It is well known that the imbalance between free radical generation and scavenging leads to oxidative stress, often causing organ dysfunction. Oxidative stress is particularly harmful to the neonatal brain due to low concentrations of antioxidants and high oxygen consumption associated with the transition from fetal to postnatal life [3,6]. The majority of ROS—such as superoxide, hydroxyl radical, hydrogen peroxide, and peroxynitrite—damage intracellular targets including proteins, lipids, DNA, and neural membranes [7]. As antioxidants can interact with ROS and interrupt free radical chain reaction before vital molecules are damaged, it seems reasonable to assume that exogenous antioxidant therapy may have the potential to reduce the associated cellular damage [8]. Indeed, antioxidant therapy of cerebral ischemia has been found to be very efficient, and in the last three decades, it has been reported that ROS-mediated neuron and astrocyte loss was prevented by antioxidants in animal models of cerebral ischemia [9].

Despite the substantial research of antioxidants effects that has been conducted regarding HIE or stroke, the therapeutic interventions remain limited. One of the limitations of the wide application of antioxidants in clinic is related to their side effects associated with violation of redox balance in cells. Maintenance of redox state homeostasis is known to be critical for cell viability, activation, and proliferation [10]. Numerous cellular signaling pathways are involved in redox regulation, and they can be blocked by antioxidants, yielding unwanted deleterious effects [11]. Thus, the targeted delivery of the antioxidants in the loci where the pathological levels of ROS are generated could make antioxidant therapy more effective. It is well-documented that mitochondria can be responsible for creating pathological levels of ROS in the brain (for instance during reperfusion after ischemia) [12,13].

The mitochondrial approach was implemented by Murphy’s group through synthesis of mitochondria-targeted conjugate of ubiquinone and vitamin E, which has been shown to alleviate certain pathological conditions [14,15]. Mitochondria-targeted antioxidative constructs contained a permeable positively charged moiety able to be electrophoretically accumulated in mitochondria, e.g., the alkyl triphenylphosphonium ion. Previously, we have shown that plastoquinone as an antioxidative part of the conjugate demonstrates wide protective effects [16] and later it has been elucidated that neuroprotective properties of mitochondria-targeted antioxidants were determined not only by the quinone part but also the penetrating part itself [17]. Plastoquinone is present in natural photosynthetic systems, being an electron carrier in electron transport chains of chloroplasts and cyanobacteria reported to possess a strong antioxidative property [18,19]. The therapeutic efficiency of mitochondria-targeted antioxidants has been demonstrated in animal models of oxidative stress-related pathologies of brain [17,20,21,22], kidneys [22,23,24,25,26], and heart [27,28]. A comprehensive clinical study of eye drops based on the mitochondrial-targeted antioxidant to treat dry eye syndrome confirmed the safety and efficiency of this class of antioxidants [29]. In spite of wide usage of mitochondria-targeted drugs in treatment of different pathologies, until recently, there have been no attempts to use them in neonatal pathologies, especially in acute cerebral disorders.

The aim of this study was to explore the neuroprotective properties of the mitochondria-targeted chimeric compound, 10-(6′-plastoquinonyl) decylrhodamine 19 (SkQR1) in neonatal hypoxic–ischemic brain injury.

## 2. Results

### 2.1. Hypoxia-Ischemia Induces Mitochondrial Dysfunction and Oxidative Stress

**Figure 1:**
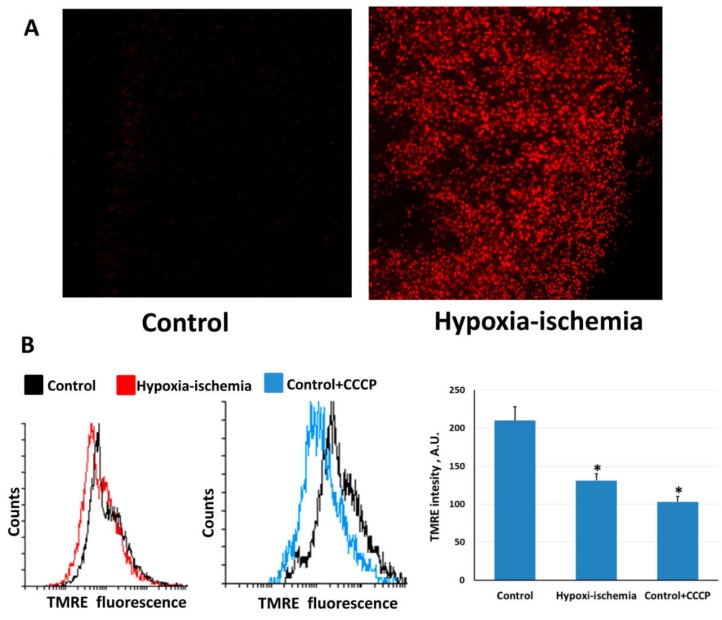
Hypoxia–ischemia increases mitochondrial ROS production in brain cells as measured by MitoSOX staining in brain slices obtained after 10 min of reoxygenation (**A**). Mitochondrial dysfunction is documented by a decreased mitochondrial membrane potential, as monitored by TMRE fluorescence (**B**). * *p* < 0.05 compared to control, one-way ANOVA, followed by Tukey’s post hoc analysis.

We used the vital brain slices for evaluation of oxidative stress in neonatal brain after hypoxia–ischemia (HI). The slices were stained with MitoSOX™ Red, a fluorogenic ROS-probe specifically targeted to mitochondria in live cells. HI caused an explosive increase of MitoSOX fluorescence in slices made from ipsilateral hemisphere after 10-min reoxygenation (Figure 1А). This indicates that mitochondria are responsible for the ROS burst associated with HI. Simultaneously, brain HI followed by restoration of oxygen supply resulted in a dramatic drop of the mitochondrial membrane potential over the brain cells (about 62% by the TMRE fluorescence intensity compared to control values, Figure 1В). The addition of 1 μM CCCP to the suspension of control brain cells resulted in a similar decrease of the mitochondrial membrane potential, which corresponds to at least partial mitochondrial deenergization as a result of HI (F[2,24] = 45.29, Figure 1B).

### 2.2. Mitochondria-Targeted Antioxidant SkQR1 Diminishes Oxidative Stress in Brain after Hypoxia–Ischemia

**Figure 2:**
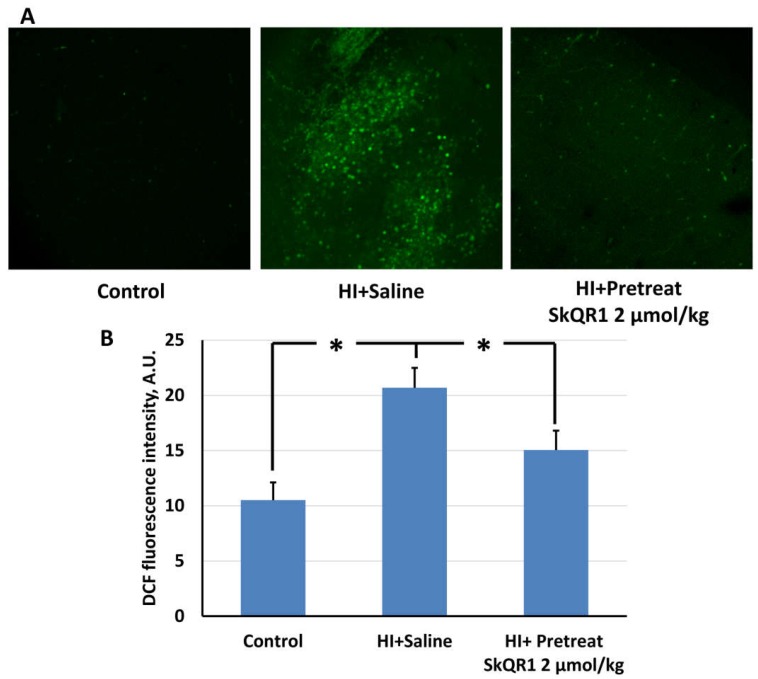
SkQR1 eliminates excessive ROS production in brain after hypoxia–ischemia. ROS levels were evaluated 10 min after reoxygenation by confocal microscopy of vital brain slices loaded with DCF-DA (**A**). Diagram represents quantitation of DCF fluorescence intensity (**B**) as mean ± SEM (*n* = 15). Pretreatment with 2 μmol/kg SkQR1 24 h prior to HI results in significantly lower DCF fluorescence in response to HI, * *p* < 0.05, one-way ANOVA, followed by Tukey’s post hoc analysis.

ROS production examined by a ROS-sensitive probe, 2′,7′-dichlorodihydrofluorescein diacetate (DCF) fluorescence in brain tissue exposed to HI was about doubled as compared to the control brain slices (Figure 2). When pups received SkQR1 intraperitoneally (2 μmol/kg) 24 h before ischemic-hypoxic challenge, ROS production associated with HI was significantly diminished (F[2,24] = 83.07, Figure 2).

### 2.3. Effects of Pretreatment with Mitochondria-Targeted Antioxidant SkQR1 on Infarct Size

**Figure 3:**
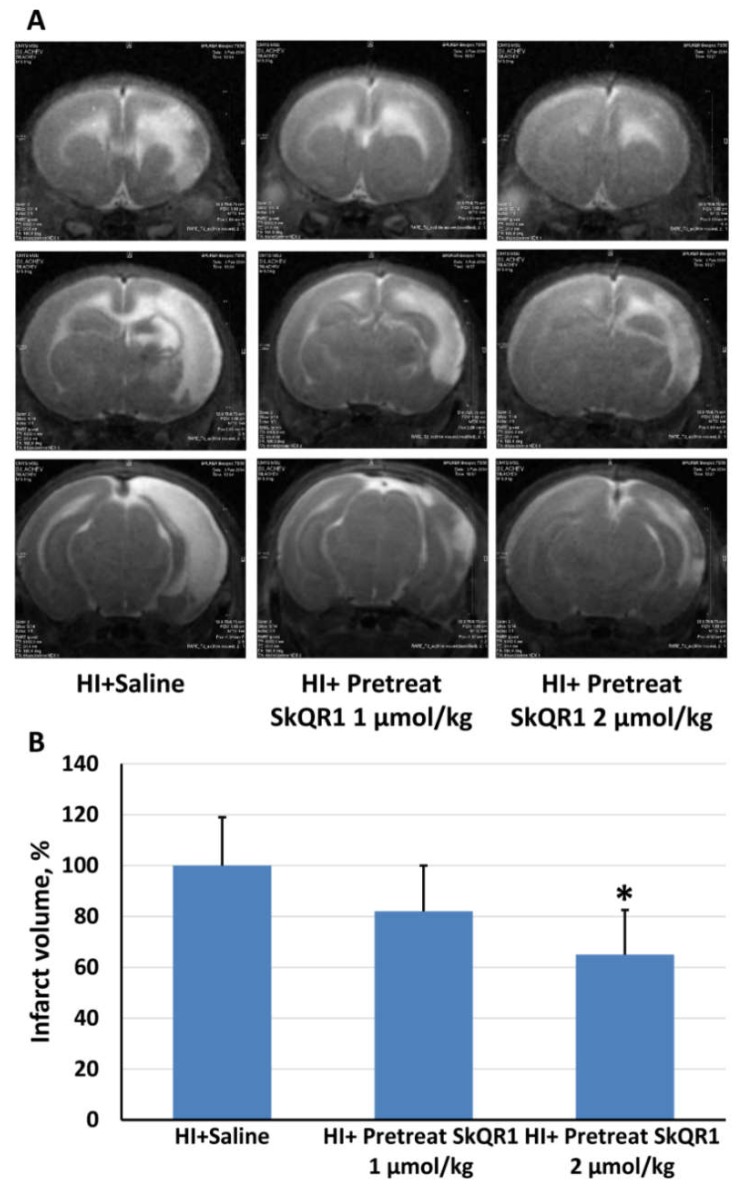
Evaluation of the neuroprotective effect of mitochondria-targeted antioxidant SkQR1 on the severity of brain damage at the seventh day after brain hypoxia–ischemia induction in rat pups. (**A**) Three representative brain sections obtained by T2-weighed MRI (each image covered 0.8 mm thick brain section). Hyperintensive regions refer to ischemic areas. (**B**) Infarct volume evaluated by using MRI with analysis of T2-weighted images. * *p* < 0.05 denotes significant difference from the HI + Saline group, one-way ANOVA, followed by Tukey’s post hoc analysis.

We extensively examined the HI-induced damage in sensorimotor cortex, striatal, and hippocampus using magnetic resonance imaging seven days after exposure of the pups brain to HI (Figure 3A). In the groups of control and sham-operated animals, no damage was detected. HI caused a remarkable damage of the brain, covering approximately 45% of the volume of the effected hemisphere. The pretreatment with SkQR1 at a dose 2 μmol/kg significantly reduced infarct volume to 65 ± 17.5% if normalized to the saline-threated HI group (F[2,29] = 3.69, *p* < 0.05, Figure 3B). As to 1 μmol/kg SkQR1, it demonstrated only a trend for a recovery from HI exposure.

### 2.4. Effects of Mitochondria-Targeted Antioxidant SkQR1 Pre- and Post-Treatment on Sensorimotor Deficits in HI Rats

**Figure 4:**
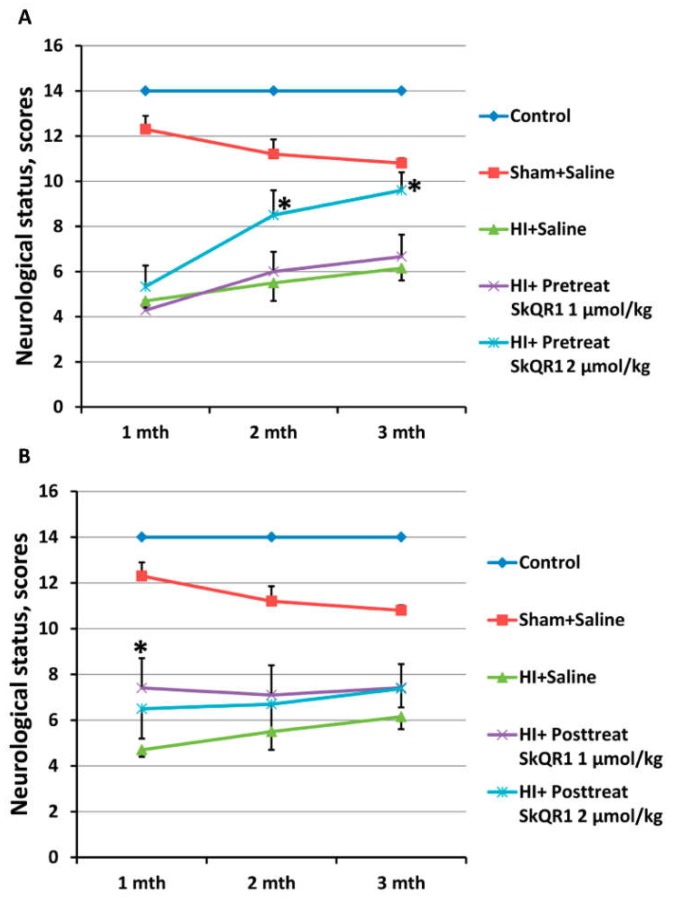
Effect of mitochondria-targeted antioxidant SkQR1 on neurological status determined by a limb-placing test on first, second, and third month after HI. SkQR1 was administrated i/p 24 h before HI (**A**) or 10 min after start of reoxygenation (**B**) at doses 1 and 2 μmol/kg. Data is expressed as mean ± SEM * *p* < 0.05 denotes significant difference from HI + Saline group (Kruskal–Wallis test with the Mann–Whitney *u*-test).

Earlier, it has been shown that assessment of the motor and sensorimotor deficiency may report on brain dysfunction in a neonatal brain hypoxia–ischemia model [30]. In the present study, we used those neurological tests which in adult animals demonstrated the highest efficiency in experiments using focal ischemia associated with a long-term sensorimotor deficiency [31]. In these experiments, the limb-placing test and the staircase test were employed. Using the limb-placing test, we found that HI of neonatal brain causes sensorimotor dysfunction of fore- and hindlimbs. During the entire experiment, control animals did not show any neurological dysfunction, and their neurological status was scored by 14 points. One month after HI, the sham-operated rats scored 12 ± 0.6 points, whereas the animals in the HI + Saline group scored only 4.7 ± 0.3 points (Figure 4A). Three months later, we observed a spontaneous decrease of neurological deficiency in the HI+Saline group, whose score rose up to 6.2 ± 0.5 points, whereas the neurological status of the sham-operated animals dropped to 11 ± 0.2 points. The pretreatment with SkQR1 at a dose 2 µmol/kg improved functional recovery as measured by neurologic status scores after two and three months to 8.5 ± 0.8 and 9.6 ± 0.8 points, respectively (*p* < 0.05, Figure 4A). There were no statistically significant differences in neurological status after treatment with 1 µmol/kg SkQR1 as compared to HI + Saline group.

**Figure 5:**
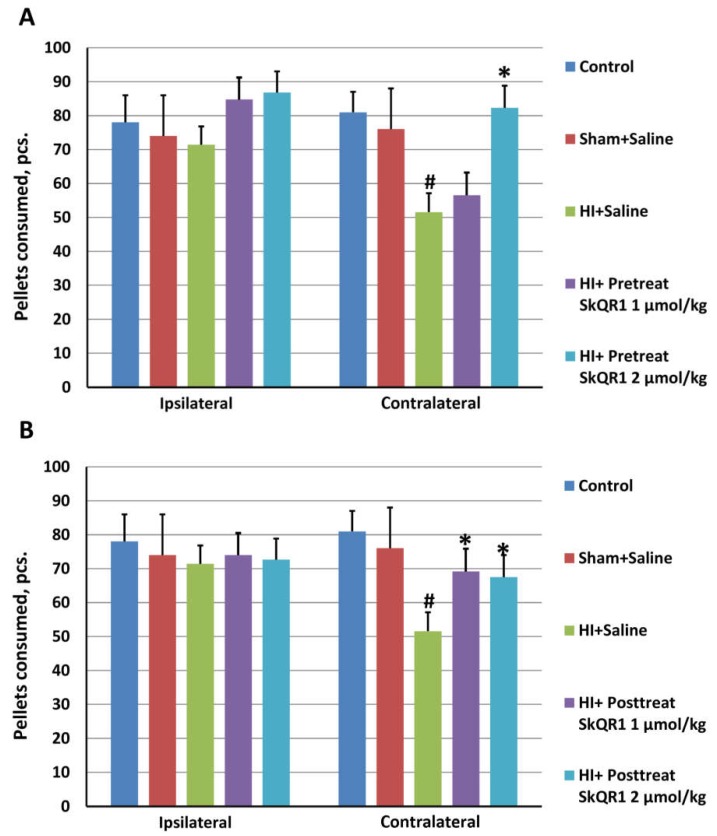
Effect of mitochondria-targeted antioxidant SkQR1 on a skilled motor task determined by the staircase test three months after the HI induction. SkQR1 was administrated i/p 24 h before HI (**A**) or 10 min after start of reoxygenation (**B**) at doses 1 and 2 μmol/kg. Results represent the amount of all consumed pellets during 10 days of testing for each side. Data are expressed as mean ± SEM. ^#^ *p* < 0.05 denotes significant difference between control with HI + Saline groups with ipsilateral side; * *p* < 0.05 denotes significant difference from HI + Saline group, one-way ANOVA, followed by Tukey’s post hoc analysis.

The staircase test was performed three months after HI modeling during 10 days. We calculated the total number of the consumed sugar pellets in the course of this period. In the control and sham-operated groups, statistically significant differences between relative to damage sides (contralateral and ipsilateral) were not detected in the number of the captured and consumed sugar pellets. The animals in HI + Saline group had a pronounced disturbance in the contralateral forelimb skilled reaching, which consisted in their inability to get the sugar pellets from the cells on the lower steps. In this group, the number of consumed pellets was significantly lower down to 25% if compared contralateral with the ipsilateral sides (*p* < 0.05, Figure 5A). Post hoc analysis showed that the rats of SkQR1 group which receive a dose 2 µmol/kg, but not 1 µmol/kg, retrieved significantly more pellets than animals from HI + Saline (F[2,27] = 8.39, *p* < 0.05, Figure 5A).

To elucidate whether post-treatment with SkQR1 was able to provide a therapeutic effect on neonatal rats following acute phase of HI, SkQR1 at doses 1 and 2 µmol/kg was administered 10 min after HI with subsequent evaluation of long-term neurological effects. Post-treatment with SkQR1 resulted in a statistically significant decrease of neurological deficit only at first month at 1 μmol/kg dose in the limb-placing test. In the next two months, we observed only a trend to sensorimotor recovery for both dosages of SkQR1 (Figure 4B). However, in the staircase test, rats treated with SkQR1 at doses 1 and 2 μmol/kg demonstrated a significant improvement of skilled motor functions (F[2,27] = 3.84, *p* < 0.05, Figure 5B).

## 3. Discussion

In spite of an intensive search for neuroprotective drugs, nowadays the only clinical relevant approach to treat neonates with HIE is hypothermia. Although it does not significantly reduce the death toll or severe disabilities, hypothermia results in improved neurologic outcome in survivors after HIE [32]. It was also shown that hypothermia attenuates oxidative stress after severe traumatic brain injury in infants and children [33]. It is believed that one of the main mechanisms of action of hypothermia is a decrease in basal metabolism and ultimately the activity of many enzymatic reactions, including those producing ROS. Thus, hypothermia can be considered as an antioxidative strategy, and therefore other approaches that diminish the levels of ROS in the brain tissue can be an effective therapeutic approach for the treatment of HI. Unfortunately, the use of hypothermia is possible only in newborns with gestational age from 32 weeks, while modern neonatology is able to care for children from 24–25 weeks gestation. Thus, it seems necessary to develop new effective therapeutic approaches, one of which may be antioxidant therapy in the acute period of hypoxia-reoxygenation.

Nowadays, many substances possessing antioxidative property show the ability to protect the neonatal brain from hypoxia–ischemia in experimental models (see reviews [34,35]). N-acetylcysteine, an oxygen radical scavenger that can restore intracellular glutathione levels, has been extensively studied in animal models of various neurological disorders [36]. In neonatal HIE rats treated with systemic hypothermia, the supplement with N-acetylcysteine reduced the volume of the brain damage at both two weeks and four weeks after the hypoxic–ischemic insult [37]. Hormones with antioxidative properties, such as melatonin or erythropoietin, displayed neuroprotective properties in HIE models [38,39,40]. However, the therapeutic mechanism may include several targets for these substances [41,42]. Nevertheless, no antioxidants have been approved for HIE treatment either by the United States Food and Drug Administration or by the European Medicine Agency. Despite this, clinical studies of antioxidants continue. It has been shown that erythropoietin, both used in monotherapy mode and also in combination with hypothermia in infants with hypoxia, has neuroprotective effect [43,44,45,46]. Available data highlights that a hypoxic–ischemic injury is a complex process, and a therapeutic effect can be obtained only using a strategy that targets multiple pathways.

**Figure 6:**
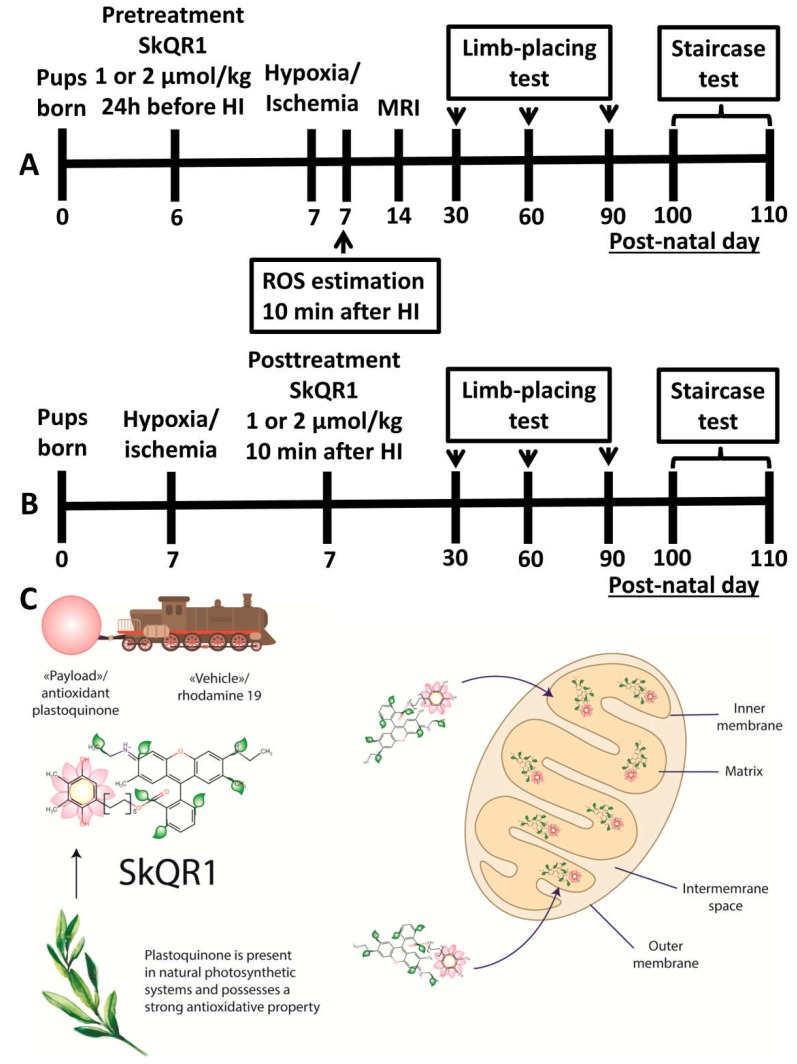
The study design. SkQR1 was administered intraperitoneally by two protocols: (**A**) pretreatment 24 h before HI or (**B**) posttreatment 10 min after reoxygenation onset at doses 1 or 2 μmol/kg. (**C**) The concept of mitochondria-targeted delivery of antioxidant SkQR1 payload into mitochondria.

Obviously, mitochondria play a crucial role in the mechanism of HI-induced brain damage as well as in the signaling protective mechanisms. Therefore, one of the targets for therapy may be those mitochondrial ROS which were produced during the hypoxic and reoxygenation phase. The electric driving force concept of antioxidant delivery to the mitochondria was first suggested by the Murphy’s group. They synthesized a compound composed of a thiobutyl residue chemically linked to a phosphonium cation [14]. Later, our group synthesized mitochondria-targeted antioxidants containing positively charged phosphonium or rhodamine moieties linked to plastoquinone by a decane linker [16]. This principle allows delivery of practically any antioxidant molecule to the mitochondrial matrix (Figure 6C). Earlier, it was established that SkQR1 can easily penetrate planar, mitochondrial, and outer cell membranes and electrophoretically be accumulated in mitochondria [16,27,47] including brain mitochondria [48]. We have chosen plastoquinone as a highly effective antioxidant molecule selected by nature over millions of years of evolution. The photosynthetic electron transport chain is the principal site of generation of ROS in plants under illumination. Due to the steady production of ROS, chloroplasts are protected by highly efficient antioxidative system [49,50]. The reduced form of plastoquinone (plastoquinol) was shown to function as an antioxidant by diminishing ROS produced during photosynthetic reactions [51]. As to the specificity of mitochondria-targeted plastoquinone toward different ROS, theoretically, due to the high redox potential of the pair redSkQR1/oxSkQR1 (+80 mV) its plastoquinone can quench superoxide anion, hydrogen peroxide, and lipid peroxides (other aggressive ROS are derivatives of these). Also, it was shown that SkQR1 demonstrates the additional antioxidative effect through mild uncoupling, i.e., through diminishing the mitochondrial membrane potential (particularly, mediated by fatty acids, see [52]), thus lowering the production of ROS by mitochondria. Such complex effects of members of SkQ variety makes us not only focus on direct interaction of SkQ with ROS, but broadly name antioxidants of a wide spectrum of action.

In this study, we explored neuroprotective properties of SkQR1, the conjugate of plastoquinone with rhodamine residue using experimental design shown in Figure 6. We found that pretreatment with SkQR1 significantly reduces brain injury, diminishing nervous tissue loss measured at 14th day after the injury and protecting neuronal networks responsible for sensorimotor functions. Ultimately, SkQR1 ameliorated HI-induced short- and long-term behavioral deficits and significantly improved sensorimotor performance at dose 2 μmol/kg. The used protocol of introduction of SkQR1 in the early period of reoxygenation confirmed its efficiency in restoring long-term skilled motor functions by two concentrations (1 and 2 μmol/kg) but not in the limb-placing test. Difference in responses using the staircase and limb-placing tests may be associated with delayed neurodegeneration effects. It has been shown that HI in the developing brain causes both early and delayed neurodegeneration in specific systems in which the morphology of neuronal death is determined by time, region, and potentially by patterns of neuronal connectivity [53]. In this regard, the key point is the time of initiation of therapy. SkQR1 therapy was started 10 min after the start of reoxygenation, when the cells were already exposed to 2 h of ischemia and 10 min of reoxygenation, which may be sufficient for irreversible damage to the cells in the nuclear zone and, accordingly, they cannot be rescued. However, such a mode of treatment can prevent delayed death of neurons forming the neural network with earlier killed cells located in different parts of the central nervous system, and thus prevent the loss of complex sensorimotor functions, including those analyzed by the staircase test [54,55]. On the other side, the limb-placing test evaluates responses to various stimuli, balance, and simple motor functions, which reflect damage to a specific area of the cerebral cortex [56].

The dose of SkQR1 2 µmol/kg was found to be the most effective, which is consistent with the data obtained in the model of focal ischemia in adult animals. We demonstrated that treatment with SkQR1 prevents motor impairments and neurological functions and significantly reduces the volume of infarct and brain edema in the middle cerebral artery occlusion as a model of stroke in rats [17,20]. However, the study of mitochondria-targeted antioxidant MitoQ at doses of 17 or 51 µmol/L directly introduced into the striatum one day before and two days after neonatal rat hypoxia–ischemia did not have any significant effect on the total number of striatal medium-spiny neurons [57]. The absence of a positive MitoQ effect can be explained on one side by a narrow concentration window between antioxidative and pro-oxidative properties of MitoQ, since the authors used 10 times higher antioxidant concentration than what we used in our study [18]. On the other hand, we have previously shown that plastoquinone conjugated with a penetrating cation alkyl triphenylphosphonium, as well as MitoQ, had no significant effect on the volume of brain damage in a rat model of stroke, while plastoquinone derivatives conjugated with rhodamine (SkQR1) was very efficient [17]. We must note that a mitochondria-directed antioxidant is a complex chimeric molecule, neuroprotective properties of which depend on both the antioxidant part and the penetrating cation.

There is little doubt about the fact that oxidative stress plays a fundamental role in early injury to the neonatal brain regardless of the gestational age. However, the question remains open: what is (are) the source(s) of the ROS production under specific conditions? This is primarily due to the methodological difficulties of studying the processes of formation of ROS considering their short time of life. The most common approach to assess oxidative stress is to evaluate secondary products obtained by interaction of ROS with proteins, nucleic acids, or lipids. It has been reported that the levels of plasma malondialdehyde, a stable product formed as a result of lipid peroxidation, were significantly higher in infants exposed to HIE [58]. This data confirms that oxidative stress takes place at HI but does not elucidate mechanisms of ROS formation. To gain better insight into the mechanism of oxidative stress development, the evaluation of ROS production using fluorescent dyes, directed to various compartments or organelles of the cell, is widely used. Fluorescent probes are reliable sensors of ROS due to their high sensitivity, simplicity in data collection, and high spatial resolution in microscopic imaging techniques [59]. In general, data on pathological formation of ROS were obtained mainly on primary cultures of neural cells in the modeling of hypoxia–ischemia. Using neuronal cultures, Abramov with co-authors showed that three distinct mechanisms contribute to neuronal injury by generating ROS and consecutive oxidative stress depending on the stage of hypoxia and reoxygenation. Thus, under hypoxic conditions, mitochondria generate an initial burst of ROS by complexes I and III of the electron transport chain. Xanthine oxidase is responsible for the second phase of ROS production during hypoxia, and its inhibition reduces the production of ROS that strongly suggests an involvement of this enzyme in oxidative stress. A third phase of ROS generation revealed at reoxygenation was attributed to the calcium-dependent activation of NADPH oxidase [60]. Thus, in the cell under pathological conditions, there is a spatial distribution of ROS generators, between which there may be a cross-talk, which makes it impractical to use one single pharmacological antioxidant. It is important to note that oxidative stress can be developed in a delayed time phase after HI. In a recent study, 38 days after HI, an increase in the oxidative stress strength was observed associated with a decrease in the activity of antioxidative enzymes such as superoxide dismutase and catalase [61]. These changes correlated with the volume of hippocampus damage and spleen weight, which indicates the involvement of inflammatory processes on the development of oxidative stress in the long-term scale after HI. Thus, inflammation-mediated oxidative stress in HIE may occur in the remote period after injury, but the produced ROS are not of mitochondrial origin [4]. However, it was shown that no signs of oxidative stress can be observed on the 86th day after HI [62]. In this context, our aim was to study the effect of a mitochondria-targeted antioxidant in one of the primary phases of production of ROS by the respiratory chain of mitochondria, which lasts for a short time [60], but it causes severe pathological consequences which is also evidenced by our results. Ultimately, it has been concluded that mitochondria are one of the major sources of ROS which trigger oxidative stress. It is also important to note that even low levels of mitochondrial ROS-induced mitochondrial permeability transition pore (mPTP) openings may result in an explosive ROS burst leading to destruction of mitochondria. This amplification cycle of mitochondrial ROS formation and release was named ROS-induced ROS release [63,64]. Using a cell suspension obtained 10 min after the start of reoxygenation and stained with the membrane potential dye TMRE, we observed a sharp drop in the mitochondrial potential, which indicated the opening of mPTP. In our experiments, this drop was accompanied by increased production of ROS evaluated by the mitochondria-targeted ROS sensor MitoSOX. In these experiments, we used vital brain slices, which have an obvious advantage of preserving the complex architectural relations between different cells in the tissue. Using the vital brain slices, for the first time we demonstrated that the introduction of SkQR1 one day before the challenge reduces the development of oxidative stress in the acute phase of hypoxia-reoxygenation. On the one hand, these results confirm the antioxidative mechanism of neuroprotective action of SkQR1, and on the other, the involvement of mitochondrial ROS in the pathogenesis of HIE. Thus, our results are consistent with studies of the antioxidant properties of SkQR1 in the in vitro system [16] and in vivo where vital sections of the rat kidney were used [23].

It should also be noted that, in other studies, administration of SkQR1 prior to the challenge caused induction of erythropoietin synthesis in the kidneys [23], which was associated with a protective effect against ischemia/reperfusion of the brain through an increase in the phosphorylated form of glycogen synthase kinase-3β (GSK-3β) [20]. This indicates the existence of additional neuroprotective mechanisms exerted by mitochondria-targeted compounds apart from their direct antioxidative action. GSK-3β located in the vicinity of the mitochondrial permeability transition pore complex is believed to be a key enzyme that the protective signaling pathways converge on [60]. This kinase plays a principal role in ischemic neuronal death and its inhibition through phosphorylation of ser-9 affords neuroprotection [5,65].

Thus, in our work, we demonstrate that ROS produced by mitochondria are an important pharmacological target for the treatment of HI-related brain damage. However, given the incidence of many patho-biochemical cascades, including those associated with the production of ROS, it is obvious that pharmacological therapy should go the way of using a variety of targets in a certain time frame for the treatment of HI-mediated deleterious consequences. Probably, usage of NADPH-oxidase inhibitors can be also desirable. Nevertheless, we believe that a single or short course of administration of mitochondrial-targeted antioxidants is sufficient for the realization of their neuroprotective action, since the acute phase of production of ROS by mitochondria after reoxygenation does not last long. Apparently, the basis of neuroprotective action of SkQR1 is a decrease in the production of mitochondrial ROS, the generation of which increases in the first hours after HI. Protective effects are manifested in both the direct neuroprotection, i.e., reduction of cell death associated with a smaller lesion focus, as well as in more remote effects on the neurological status of animals that experienced small impairments of motor functions of the brain in adulthood. A clinical study of SkQR1 seems promising.

## 4. Materials and Methods

### 4.1. Use of Animals

The animal protocols used in this work were evaluated and approved by the institutional animal ethics committee in accordance with FELASA guidelines. The experiments were performed on outbred white rats. The animals were obtained from the animal facility of the A.N. Belozersky Institute of Physico-Chemical Biology. Dams and their pups were kept in cages with a temperature-controlled environment (23 ± 2 °C) with light on from 9:00 a.m. to 9:00 p.m. Dams had ad libitum access to food and water, and pups were checked daily for health. For long-term survival and behavioral testing, the pups were weaned on P30, and five or six animals were housed (split by sex) per cage until they were tested for neurological function.

### 4.2. Induction of Neonatal HI Animal Model and Treatment with SkQR1

We used the modified Rice–Vannucci rat model of hypoxic–ischemic brain injury [66]. Postnatal seven-day-old pups were used to induce neonatal HI with unilateral hypoxic–ischemic cerebral damage via permanent occlusion of the right common carotid artery through its annealing under isoflurane anesthesia followed by 120 min incubation at 8% oxygen, 35 °C. The mortality rate after HI induction procedure was 3–5%. Pups from at least six litters were randomly divided into the following experimental groups (Figure 6): (1) Control (*n* = 6), intact rats; (2) Sham + Saline (*n* = 6), sham-operated pups that underwent anesthesia and incision only and which received intraperitoneal (i/p) saline injection; (3) HI + Saline (*n* = 10), pups with HI induction that received i/p saline; (4, 5) HI + pretreat SkQR1 (*n* = 11), animals pretreated with SkQR1 by i/p injection 24 h before HI at a dose 1 or 2 μmol/kg; (6, 7) HI + posttreat SkQR1 (*n* = 12), animals post-treated with SkQR1 by i/p injection 10 min after HI at a dose 1 or 2 μmol/kg. This dosing SkQR1 was based on a prior study in rat stroke model [17]. The SkQR1 solution was prepared 10 min prior to administration to the animals. An aliquot of 10 mM stock solution of SkQR1 in ethanol was dissolved in saline to final concentration of 100 or 200 μM. All animals received one i/p injections in a volume of 0.01 mL/g. Control groups of animals received an equal volume of saline.

### 4.3. MRI Studies of the Brain Damage

Infarct volume was quantified by analyzing brain MRI images obtained seven days after the HI as described previously [67] on a 7T magnet (Bruker BioSpec 70/30 USR, Bruker BioSpin, Ettlingen, Germany) using 86 mm volume resonator for radio frequency transmission and a phased array mice head surface coil for reception. Before scanning, the animals were anesthetized with isoflurane (2% induction, 1.5% maintenance) in a mixture of oxygen and air. Rats were placed in a prone position on a water-heated bed. The heads of the rats were immobilized using a nose mask and masking tape. The imaging protocol included a T2-weighted image sequence (time to repetition = 4500 ms; time to echo = 12 ms; slice thickness = 0.8 mm). Ischemic damage volume for each group was normalized to the mean for the group HI + Saline.

### 4.4. The Staircase Test

At three months of age, skilled forelimb usage was measured with a staircase test. The device was developed by Montoya et al. to assess independent forelimb use in skilled reaching and grasping tasks [68]. Rats were fed with a mildly food-restricted diet during training and the test period, resulting in about a 10% reduction in body weight. Training and testing were performed by placing three food pellets (each of ≈50 mg) on each step of the staircase. Habituation of the animal to the apparatus was started 95 days after HI and lasted five days with exposure for 15 min each day. Testing started at 100 days using two 10-min sessions per day with a 2 h interval for two weeks (Monday to Friday). After each session, the number of pellets consumed was counted. Data is presented as the amount of all consumed pellets during 10 days of testing for each side of the animal.

### 4.5. Limb-Placing Test

The modified version of the limb-placing test consisting of seven tasks was used to assess forelimb and hindlimb responses to tactile and proprioceptive stimulation [69]. Rats were handled three days before testing. The limb-placing test was used to evaluate neurological deficit in animals at ages one, two, and three months. For each task, the following scores were used: 2 points, normal response; 1 point, delayed and/or incomplete response; 0 points, no response. The mean score was evaluated over seven tasks.

### 4.6. ROS Determination in Brain Slices

The intact pups or pups of groups HI + Saline and HI + Pretreat SkQR1 2 μmol/kg 10 min after start of reoxygenation were anaesthetized by i/p injections of 300 mg/kg (12%) chloral hydrate, decapitated and the brains were rapidly removed and placed in ice-cold DMEM medium in a petri dish on ice. Brain tissue slices ~100 μm thick were cut with Vibroslice vibrating microtome (WPI, Sarasota, FL, USA). During the sectioning procedure the brain tissue was immersed in ice-cold DMEM medium. Brain slices were loaded with 10 μM DCF-H2 DA or 5 μM MitoSOX™ Red (Thermo Fisher Scientific, Hampton, NH, USA) for 10 min at room temperature in the dark followed by washing the residual dye out twice in DMEM and imaging with an inverted confocal microscope. There were three rats in each group, at least three brain sections were examined from each animal.

### 4.7. Confocal Microscopy

The brain slices were imaged using a LSM510 laser scanning confocal microscope (Carl Zeiss, Oberkochen, Germany) in a multi-channel mode as appropriate for the dyes loaded. Experiments were carried out at 25 °C. Analysis of fluorescence was performed in glass-bottom dishes with the excitation at 488 nm and 543 nm and emission collected at 500–530 nm and <560 nm, correspondingly. To minimize the contribution of photo-induced mitochondria/cell damage in the relative fluorescence intensities, we analyzed images used by averaging the first four scans only. Images were processed by ImageJ software (W. Rasband, NIH, Bethesda, MD, USA, http://rsb.info.nih.gov/ij).

### 4.8. Assessment of Changes in Mitochondrial Transmembrane Potential

The MUSE™ cell analyzer (Millipore, Billerica, MA, USA) was used for measurement of changes in mitochondria membrane potential (∆ψ). The intact pups and pups exposed to HI and 10-min reoxygenation were anaesthetized by i/p injections of 300 mg/kg (12%) chloral hydrate and decapitated; the brains were rapidly removed and placed in DMEM medium at room temperature in a Petri dish. The ipsilateral hemispheres were dissected, the meninges were removed, and the tissue was incubated for 15 min in trypsin/EDTA (0.05%/0.02% wt/vol in phosphate-buffered saline (PBS) at 37 °C. The cells were rinsed twice with PBS and once with DMEM medium without bicarbonate, dissociated by Pasteur pipette in DMEM, pelleted by centrifugation (210 g for 2 min at 21 °C), and resuspended in DMEM. Cell suspension containing 1 × 10^5^ cells was divided by two samples: unstained cells used as a negative control and those stained with TMRE. Cell suspension was stained with 100 nM TMRE for 10 min at room temperature. To wash out the excessive dye, cells were centrifuged (210 g for 2 min at 21 °C) and resuspended in DMEM. For estimation of zero point of ∆ψ (completely uncoupled mitochondria), 10 min prior to staining with TMRE, the uncoupler (1 µM СCCP) was added to cell suspension made from brain of intact animals and was kept during TMRE loading. A 543-nm laser excitation was used, and emission beyond 560 nm was collected. Totally, 10,000 cells per sample were taken; each experiment included usage of three animals.

### 4.9. Statistics

Statistical analyses were performed using STATISTICA 7.0 for Windows (StatSoft, Inc., Palo Alto, CA, USA). Values are given as mean ± standard error of the mean (SEM). Variance homogeneity was assessed with Levene’s test. Statistical differences in TMRE and DCF intensity, infarct volume and pellets consumed between the groups were analyzed using one-way ANOVA with Tukey’s post hoc test. Statistical differences in the limb-placing test scores between the groups were analyzed using the Kruskal–Wallis test with the Mann–Whitney *u*-test (the Bonferroni post hoc correction was applied). Differences were considered significant at *p* ≤ 0.05.
